# Stability of glycoprotein gene sequences of herpes simplex virus type 2 from primary to recurrent human infection, and diversity of the sequences among patients attending an STD clinic

**DOI:** 10.1186/1471-2334-14-63

**Published:** 2014-02-06

**Authors:** Lars Haarr, Arvid Nilsen, Per M Knappskog, Nina Langeland

**Affiliations:** 1 Department of Clinical Science, The Faculty of Medicine and Dentistry, University of Bergen, Bergen, Norway; 2 Department of Clinical Medicine, The Faculty of Medicine and Dentistry, University of Bergen, Bergen, Norway

**Keywords:** Primary and recurrent infections of humans, Genetic stability of HSV-2

## Abstract

**Background:**

Herpes simplex virus type 2 (HSV-2) is sexually transmitted, leading to blisters and ulcers in the genito-anal region. After primary infection the virus is present in a latent state in neurons in sensory ganglia. Reactivation and production of new viral particles can cause asymptomatic viral shedding or new lesions. Establishment of latency, maintenance and reactivation involve silencing of genes, continuous suppression of gene activities and finally gene activation and synthesis of viral DNA. The purpose of the present work was to study the genetic stability of the virus during these events.

**Methods:**

HSV-2 was collected from 5 patients with true primary and recurrent infections, and the genes encoding glycoproteins B,G,E and I were sequenced.

**Results:**

No nucleotide substitution was observed in any patient, indicating genetic stability. However, since the total number of nucleotides in these genes is only a small part of the total genome, we cannot rule out variation in other regions.

**Conclusions:**

Although infections of cell cultures and animal models are useful for studies of herpes simplex virus, it is important to know how the virus behaves in the natural host. We observed that several glycoprotein gene sequences are stable from primary to recurrent infection. However, the virus isolates from the different patients were genetically different.

## Background

Herpes simplex viruses (HSV) are widely distributed pathogens transmitted by close contact. Infection by type 1 (HSV-1) starts in childhood and increases during the following years so that more than 70% of the population is seropositive at an age of 40 years [1]. HSV-1 affects the orofacial region. Additionally it has become a common cause of genital herpes infections, presently responsible for at least 50% of the cases in some regions [2,3]. Encephalitis due to HSV-1 infection is a much more rare, but devastating disease [4-7].

Herpes simplex type 2 (HSV-2) is sexually transmitted, leading to blisters and ulcers in the anogenital region (for review, see ref. [8,9]). Prevalence of HSV-2 infections shows geographical variation and marked differences from one demographic group to another (reviewed in 1). It is high among prostitutes [10,11], related to number of sexual partners and to exposure to other sexually transmitted diseases [12,13]. The average prevalence is 17% in USA [14] and varies between 7% and 31% among European adults [1]. There is a well-documented relationship between infections with HSV-2 and HIV-1 [12,15-17]. HSV-2 seropositivity increases the risk for HIV acquisition by a factor of 3 [15].

Together with varicella zoster virus (VZV) and pseudorabies virus (PRV), HSV-1 and HSV-2 belong to the *alphaherpesvirinae subfamily* of *herpesviridae*. They are all neurotropic, and like many other members of the *herpesviridae* family cause latent infections. After primary infection in the skin/mucosal membranes the virus enters axons of innervating neurons, migrates retrograde and establishes latent infections in neuronal cells in sensory ganglia. Reactivation from latency is a commonly occurring event during which the virus migrates anterograde in the axon, leading to new infection in the periphery or to asymptomatic shedding of the virus. About 90% of the persons with primary HSV-2 infection have recurrence during the first year afterwards, and one fifth of them more than 10 times during this period [18-20]. Reactivation of HSV-2 is most frequent during the first years after primary infection. As much as 75% of these events may be short asymptomatic shedding lasting for approximately 12 hours [19].

The molecular processes involved during the various steps of primary and recurrent infection have been studied extensively and reviewed in recent reports [21,22]. Neither virus particles nor viral antigens are produced in the latent state, but latency-associated transcripts (LATs) and several micro RNAs are formed [23-27].

Major regulatory steps in these events are silencing of viral genes, continuous suppression of gene activities and finally stimulation of such activities followed by DNA synthesis. Several aspects of these processes have been studied in cell culture and in animal models, but with little focus on the genetic stability of the virus. The purpose of the present work was to study this stability, in the natural host, during these different series of events. We isolated HSV-2 from true primary and recurrent infections and sequenced a set of selected genes. The isolates were genetically different, but no sequence differences were observed between the initial infection and the reactivation.

## Methods

### Clinical HSV-2 isolates

HSV-2 isolates were collected from patients with genital lesions attending a clinic for sexually transmitted diseases in Bergen, Norway. Blood samples were analysed for the presence of HSV-2 antibodies as described previously [28]. Briefly, an oligopeptide corresponding to an antigen region in glycoprotein G of HSV-2 (gG-2) was used in an enzyme-linked immunoabsorbent assay (ELISA). Patients with primary infection were selected on the basis that there was no information of previous genital infection, and HSV-2-specific antibodies were not detected when presenting with blisters and/or ulcers for the first time. Five persons were included in the study. Description of them is given in Table 1. The patients were examined again at the first reactivation of genital herpes. Sterile Dacron swabs were used to collect lesional specimens. The swabs were stored in liquid virus transport medium. Virus from primary infection was confirmed as HSV-2 by using nested PCR targeting the type-specific promoter region of the gD-2 gene as described by Cinque et al. [29] and slightly modified [30]. The clinical isolates were analysed further at a low passage number (less than 5).

**Table 1:** Patients included in the study

Patient no^*^	**Time between primary and recurrent infection**	**Treatment (valaciclovir)**	
		**Primary infection**	**Recurrent infection**
1	4 months	Yes	No
2	2 weeks	Yes	Yes
3	3 weeks	Yes	No
4	3 months	Yes	Yes
5	6 weeks	Yes	Yes

*All patients were women between 19 and 35 years old.

### Ethics and consent statement

The study was approved by the Regional Committee for Medical and Health Research Ethics, Western-Norway, University of Bergen. All patients were informed about the various aspects of the project, including isolation of virus from the samples, analysis of the viruses and publication of the results. The samples and results were coded to by anonymous. Consent was obtained from all patients.

### DNA isolation, PCR amplification and sequencing

Virus DNA was isolated using the Qiagen DNeasy Tissue Kit, as described by the manufacturer. The concentration varied from 25 to ≥ 1200 ng per μl. Three regions of the HSV-2 genome were amplified prior to sequencing. The UL27 gene (encoding glycoprotein B, gB-2) was amplified as a 2942-bp fragment spanning the region from 78 bp upstream of the start codon to 149 bp downstream of the termination codon (the positions refer to strain HG52). Several additional primers were used for sequencing (Table 2). Amplification of the US4 gene (encoding glycoprotein G, gG-2) and a fragment containing both the US7 and US8 genes (encoding glycoproteins gI and gE, gI-2 and gE-2, respectively) as well as noncoding sequences between the two genes were performed as described previously [31]. The fragment including the US4 gene contained 57 bp upstream and 39 bp downstream of the coding sequences. Similarly, the latter fragment contained 57 bp upstream of the start codon of the US7 gene and 47 bp downstream of the US8 gene. The primers used for amplification and sequencing of the two latter fragments have been published [31,32].

**Table 2:** Primers used for amplification and sequencing of the gB gene

**Nucleotide position**	**Type**	**Sequence**
**53254**–**53273**	**AS**	**5**′-**CCGTTAGCACATGTCTGCAT**
53437–53456	S	5′-AGGTACTCTCCGCTCCACAA
53522–53541	AS	5′-TCTTTCTGGCCTTGTGTTCC
53710–53739	S	5′-CTACGTCCTGCAACTGCAAC
53801–53820	AS	5′-CGAAGGGGTTGGACATAAAG
54013–54032	S	5′-CTGCTGGACTACACGGAGGT
54108–54128	AS	5′-AGGTCGATGAAGGTGCTGAC
54299–54319	S	5′-GCTTTCGGTACGAAGACCAG
54371–54390	AS	5′-AGTTCTGCACGATCACGTTG
54510–54529	S	5′-ACCACGAGCTGACTCTCTGG
54730–54749	AS	5′-TACTCCCGCACGTACAGCTC
54943–54962	S	5′-ATCTCGACCACCTTCACCAC
55027–55046	AS	5′-CACTTGGTCATGGTGCAGAC
55232–55252	S	5′-TTGTGTACATGTCCCCGTTTT
55372–55391	AS	5′-TACTTGAGGTCGGTGGTGTG
55481–55500	S	5′-CAAGTACGTGCGGAACAACA
55643–55662	S	5′-CAAATTCAAGGCCACCATGT
55648–55667	AS	5′-GTGGCCTTGAATTTGTACGG
55914–55923	AS	5′-CTTTTTGGTTTTCCGCTTCC
55914–55923	S	5′-GGAAGCGGAAAACCAAAAAG
**56176**–**56195**	**S**	**5**′-**CCATCCTCTACTCGGTCCTG**

Nucleotide positions are given for the reference strain HG52, GenBank accession number Z 86099.2. Boldface indicates primers used for amplification, the rest were used for sequencing. S: sense. AS: antisense.

PCR and gel electrophoresis of the amplified fragments were performed as described previously [30]. Briefly, Tfl DNA polymerase (Epicentre, Madison, USA) and buffer solution GN (Epicentre, Madison, USA) were used in a total volume of 50 μl containing 5 μl of diluted, purified DNA extract. The incubation steps were 5 min initial denaturation at 96°C, 30 cycles of denaturation at 95°C for 1 min, annealing of primers for 1 min at 57°C for the US7-US8 fragment or at 60°C for the UL27 and US4 fragments, elongation for 3 min at 68°C and a final extension cycle at 68°C for 15 min.

Sets of overlapping primers as shown in Table 2 and elsewhere [31,32] were used for sequencing. PCR products were treated with Exo-SAP (Affymetrix, Ca. USA) and sequenced using the Big-Dye kit ver.3.1 (Life Tech., Ca. USA) Unincorporated dyes were removed using the Big-Dye X-terminator purification kit (Life Tech., Ca. USA) and the samples were analysed on an ABI 3730 sequencer (Life Tech., Ca. USA).

### Sequence analysis

The sequences were analysed using the SeqScape software (Life Tech., Ca. USA), using JN561323.1 in the NCBI GenBank as a reference sequence. Sequences for the reference strain and for the clinical isolates were converted to FASTA (http://www.ebi.ac.uk/cgi-bin/readseq.cgi) and alignment performed using Clustal W2 (http://www.ebi.ac.uk/Tools/msa/clustalw2/). Both programs are connected to the EMBL-EBI database.

The genome of HSV-2 HG52 has two accession numbers in the GenBank, Z 86099.2 and JN 561323.2, respectively. The latter was submitted more recently than the former. Since previous published primers were also used in the present study, and their nucleotides were numbered with reference to Z 86099.2, the same reference was used for the new primers (Table 2). However, when comparing the database sequences of various clinical isolates with the reference strain, accession number JN 561323.2 was used.

## Results and discussion

To analyse for potential variation of the virus from primary to recurrent infection, we focused on genes reported to show polymorphism. They encode glycoprotein B (gB), glycoprotein G (gG), glycoprotein I (gI) and glycoprotein E (gE), respectively. The nucleotide diversity of these genes can be studied by comparing the sequences of clinical isolates of HSV-2 with the reference strain HSV-2 HG52. In the NCBI GenBank each of these genes have been sequenced in a number of isolates, varying from 48 to 69. The results of this comparison are shown in Table 3. Substitutions affecting 2 isolates or more are specified.

**Table 3:** **Sequence differences between HSV**-**2 HG52 and clinical isolates of HSV**-**2 in the database**

**Gene**	**UL27/gB (2715nt)**			**US4/gG (2100nt)**			**US7/gI (1119nt)**			**US8/gE (1638nt)**		
Total no. of isolates	69			64			49			48		
Nucleotide diversity (%) affecting:												
1 isolate	1.14			2.24			1.70			1.40		
≥2 isolates	0.81			1.52			0.80			0.55		
	***Position***	***No. of isolates***	***Substitutions***, ***comments***	***Position***	***No. of isolates***	***Substitutions***, ***comments***	***Position***	***No. of isolates***	***Substitutions***, ***comments***	***Position***	***No. of isolates***	***Substitutions***, ***comments***
	19	21	A ⇒ G	104	58	G ⇒ A	39	5	C ⇒ T	131	46	ins. GGC CGG AGG
	64-72 or 76-84*	66	del. GCC CCG GCG or GCG GCC CCG	274	26	T ⇒ C	338	2	A ⇒ G	341	2	G ⇒ T
	104	2	G ⇒ A	329	17	G ⇒ A	530	2	C ⇒ T	392	11	T ⇒ G
	106	2	G ⇒ A	405	8	C ⇒ T	618	3	A ⇒ G	605	6	C ⇒ A
	117	6	C ⇒ G	432	22	G ⇒ C	642	2	A ⇒ C	1146	8	A ⇒ G
	146	48	G ⇒ A	611	10	C ⇒ T	643	13	C ⇒ T	1211	14	A ⇒ C
	179	13	A ⇒ G	635	13	G ⇒ A	644	6	C ⇒ T	1245	2	C ⇒ T
	210	8	C ⇒ T	872	3	A ⇒ G	716	45	T ⇒ G	1293	2	C ⇒ T
	211	13	A ⇒ G	878-880	16	del. TCG	717	11	A ⇒ C	1621	44**	C ⇒ T
	850	2	C ⇒ T	891	4	G ⇒ A						
	989	16	G ⇒ A	930	50	C ⇒ T						
	1186	35	G ⇒ C	982	2	G ⇒ A						
	2247	47	A ⇒ C	993	2	G ⇒ T						
Specification of substitutions affecting ≥2 isolates	2533	29	G ⇒ C	1045	19	G ⇒ A						
				1048	64	A ⇒ G						
				1116	64	A⇒ G						
				1125	2	C ⇒ A						
				1268	37	T ⇒ C						
				1282-1285 or 1284-1286*	63	del.GCG or GGC						
				1324	7	T ⇒ C						
				1419	2	A ⇒ G						
				1470	7	G ⇒ A						
				1510	3	A ⇒ G						
				1722	2	G ⇒ T						
				1758	2	T ⇒ C						
				1761	7	G ⇒ C						
				1853	4	G ⇒ A						
				1994	2	T ⇒ G						

*Alternative alignments. **Lacking sequences in the 3′- end of a few isolates. *Nt* nucleotides, *Del* Deletion, *Ins* insertion. Positions are numbered from the first nucleotide in the start codon.

A consistent feature was that whenever a substitution was observed at a given position, it was identical in all isolates affected, regardless of the number of isolates subjected to change. This was the situation for all 4 genes. Except for deletion of 9 nucleotides in UL27 (gB), and of three nucleotides at two different positions in US4 (gG), the remaining changes were single nucleotide substitutions. Reiteration of sequences may cause alternative alignment, as observed both for the gB and the gG gene. Thus, alternative positions for substitutions may be indicated by the computer. From positions 62 to 88 in the former gene the sequence CGG CGG CCC is repeated three times. Similarly, CGG is repeated three times from position 1277 to 1285 in the gG gene. Identical variations in the same position in two or more clinical isolates indicate recombination. This was observed at numerous sites in all genes and is consistent with the fact that the isolates are from studies demonstrating different clades of virus and recombination [31,33]. However, neither sequences nor positions for nucleotide substitutions were given in these reports. In addition to substitutions generated by recombination, a number of single nucleotide substitutions affected only one clinical isolate. Such changes could be generated by random mutations or by recombination, but one cannot distinguish between these possibilities.

Nucleotide diversity affecting one isolate only varied from 1.14% (UL27/gB) to 2.24% (US4/gG). Diversity affecting two isolates or more was lower, but still highest for the US4 gene. These results indicate that the 4 selected genes might be suitable candidates for detection of potential genetic variation among viruses included in the present study.

All viral isolates were from females between 19 and 35 years old (Table 1). The time between collection of the first virus sample (primary infection) and the second one (recurrent infection) varied between 2 weeks and 4 months. All patients received treatment with valaciclovir at the first episode and 3 of them also at the second one. One patient might have had a recurrent infection between the episodes without attending the hospital clinic.

**Figure 1:**
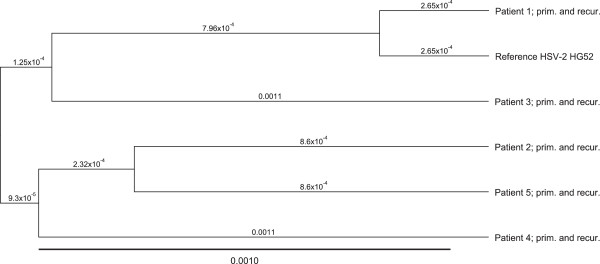
**Genetic relationship between the viruses included in the study.** The tree is based upon the open reading frame nucleotide sequences of the genes encoding gB, gG, gI and gE.

The four glycoprotein genes were sequenced in all HSV-2 isolates. Sequencing spanned the entire open reading frames and a variable number of nucleotides in the 5′- and 3′- untranscribed regions of each gene. When analysing the results from each patient separately, comparing sequences from primary to recurrent infection, no difference was detected in any patient, as indicated in Figure 1. One might not expect a substantial variation due to mutation, since proof reading during synthesis of HSV-DNA has been reported to be efficient and the rate of nucleotide substitution estimated to be 3 × 10^-8^ per site per year [34]. Similar genetic stability of HSV-2 has been observed under other conditions. Terhune et al. [35] studied isolates of HSV-2 propagated in cell culture and did not observe sequence variation in any of the genes encoding gB, gC and gD, respectively. Figure 1 also shows the genetic relationship between the viruses isolated from the 5 patients. All 5 isolates are clearly different.

There was no indication of recombination, but we cannot exclude the possibility that it might have occurred, as discussed below. For recombination to occur, at least two different viruses have to infect the same host. Such infection could be in previously uninfected sensory neurons since only a small portion of neurons harbour virus DNA in the latent state. For HSV-1 this has been shown to be between 2% and 11% [36]. However, two different viruses could also infect a single neuron, as shown for HSV-1 and VZV [37]. Since reactivation of HSV-2 is a commonly occurring event, several of them being asymptomatic [18-20], the patients could repeatedly be exposed to HSV-2 during the study, through their partners. The partners were not included in the study. Absence of detectable recombination could suggest that, in a given couple, the same type of virus was present at all times. Alternatively, the dose of a second virus could have been too low to establish a new infection [38].

All genes in the present work encode glycoproteins present in the viral envelope. Among their important roles are involvement of gB in binding virus to the cell membrane, and gE and gI acting in axonal transport of capsids and/or virions [21]. Since gG of HSV-2 is larger than that of HSV-1, it has been used as a tool for serodiagnosis of HSV-2 infection [28]. The total number of nucleotides in the open reading frames of these genes is approximately 5% of the total HSV-2 genome. Thus, the possibility should be left open that sequence variation, including recombination, might have occurred in the period from primary to recurrent infection in parts of the viral genome outside the analysed portions.

## Conclusions

Although infections of cell cultures and animal models are very useful for studies of herpes simplex virus (and other viruses), it is important to know how the virus behaves in the natural host. We have studied lesional virus isolates from humans with genital HSV-2 infection and observed that several glycoprotein gene sequences are stable from primary to recurrent infection. However, isolates from the different patients were genetically different.

## Competing interests

The authors declare that they have no competing interests.

## Authors’ contributions

AN collected blood samples and lesional material from the patients. LH was responsible for growing virus. Sequencing was a collaboration between PK and LH. NL was involved in design of the study and further discussions, and in the preparation of the manuscript. All authors read and approved the final manuscript.

## Pre-publication history

The pre-publication history for this paper can be accessed here:

http://www.biomedcentral.com/1471-2334/14/63/prepub
